# MCCM: multi-scale feature extraction network for disease classification and recognition of chili leaves

**DOI:** 10.3389/fpls.2024.1367738

**Published:** 2024-05-28

**Authors:** Dan Li, Chao Zhang, Jinguang Li, Mingliang Li, Michael Huang, You Tang

**Affiliations:** ^1^ Electrical and Information Engineering College, Jilin Agricultural Science and Technology University, Jilin, China; ^2^ School of Information and Control Engineering, Jilin Institute of Chemical Technology, Jilin, China

**Keywords:** MCCM convolutional neural network, MSFFM, MCSAM, transfer learning, chili leaf disease recognition website

## Abstract

Currently, foliar diseases of chili have significantly impacted both yield and quality. Despite effective advancements in deep learning techniques for the classification of chili leaf diseases, most existing classification models still face challenges in terms of accuracy and practical application in disease identification. Therefore, in this study, an optimized and enhanced convolutional neural network model named MCCM (MCSAM-ConvNeXt-MSFFM) is proposed by introducing ConvNeXt. The model incorporates a Multi-Scale Feature Fusion Module (MSFFM) aimed at better capturing disease features of various sizes and positions within the images. Moreover, adjustments are made to the positioning, activation functions, and normalization operations of the MSFFM module to further optimize the overall model. Additionally, a proposed Mixed Channel Spatial Attention Mechanism (MCSAM) strengthens the correlation between non-local channels and spatial features, enhancing the model’s extraction of fundamental characteristics of chili leaf diseases. During the training process, pre-trained weights are obtained from the Plant Village dataset using transfer learning to accelerate the model’s convergence. Regarding model evaluation, the MCCM model is compared with existing CNN models (Vgg16, ResNet34, GoogLeNet, MobileNetV2, ShuffleNet, EfficientNetV2, ConvNeXt), and Swin-Transformer. The results demonstrate that the MCCM model achieves average improvements of 3.38%, 2.62%, 2.48%, and 2.53% in accuracy, precision, recall, and F1 score, respectively. Particularly noteworthy is that compared to the original ConvNeXt model, the MCCM model exhibits significant enhancements across all performance metrics. Furthermore, classification experiments conducted on rice and maize disease datasets showcase the MCCM model’s strong generalization performance. Finally, in terms of application, a chili leaf disease classification website is successfully developed using the Flask framework. This website accurately identifies uploaded chili leaf disease images, demonstrating the practical utility of the model.

## 1 Introduction

According to the statistical data released by the Food and Agriculture Organization (FAO), the global chili production has shown a consistent growth trend from 1970 to 2021 (Chen et al., 2023). Studies have indicated a close correlation between chili consumption and human obesity rates as well as cardiovascular diseases (Spence, 2019). Presently, diseases affecting chili leaves have a direct impact on the production and quality of chilis. These leaf diseases typically manifest most prominently during the early stages of leaf growth. The most common method for identifying chili leaf diseases remains manual inspection of plants by qualified professionals. However, this approach is time-consuming, subjective, inefficient, and associated with high costs, and is also influenced by the expertise level of the inspector. Furthermore, existing classification strategies for chili leaf diseases have certain limitations in dealing with the diversity and large quantity of disease types. Therefore, the development of a method capable of accurately, rapidly, and efficiently identifying and classifying chili leaf diseases is of paramount importance.

In response to the aforementioned challenges, researchers have utilized traditional machine learning algorithms, including K-Nearest Neighbors (KNN) (Liang et al., 2021), Support Vector Machine (SVM) (Gangsar and Tiwari, 2019), Decision Trees (Kotsiantis, 2013), Random Forest (RF) (Gold et al., 2020), Naive Bayes (NB) (Mondal et al., 2017), and Artificial Neural Networks (ANN), to enhance feature extraction and classification algorithms (Wang et al., 2018). However, the research results indicate that while classical machine learning methods perform well in most classification tasks, they still exhibit limitations, sub-optimality, and challenges in addressing the complex issue of chili leaf diseases affected by various concurrent factors. Moreover, the complexity of managing extensive leaf disease datasets and the presence of multiple diseases further exacerbate the challenges, thereby complicating the formal deployment and application of the model.

With the continuous advancement of artificial intelligence theory, deep learning has emerged as a pivotal tool in addressing intricate visual tasks within the agricultural domain. Researchers have extensively explored various deep learning algorithms, notably Convolutional Neural Networks (CNN), to discern critical disease symptoms that significantly impact crop yield. CNN, recognized as one of the most promising technologies, has been effectively deployed for precise identification of crop leaf diseases (Saleem et al., 2019). Additionally, various models of Convolutional Neural Networks have been intricately integrated with the recognition of crop leaf diseases (Koirala et al., 2019; Van Klompenburg et al., 2020; Abade et al., 2021). For instance, Gu et al. achieved remarkable success in automatically detecting and assessing the severity of bacterial spot disease in chilis through an ensemble neural network based on CNN, attaining an impressive overall accuracy of 95.34%. Subsequently, they proposed several deep learning models employing transfer learning-based deep features for diagnosing image-based diseases and pests in chilis (Wu et al., 2020). Mathew and Mahesh utilized YOLOv5 to identify symptoms of bacterial spot disease evident on bell chili leaves sourced from farms (Mathew and Mahesh, 2022). Moreover, they specifically employed YOLOv3 to detect bacterial spot disease in bell chili plant images, achieving an average precision of 90% (Mahesh and Mathew, 2023). Mustafa et al. presented an approach based on a five-layer CNN model to automatically detect diseases in bell chili plants using leaf images, achieving an impressive accuracy of 99.99% in predicting whether the plant leaves were healthy or infected with bacteria (Mustafa et al., 2023). Furthermore, Chaitanya et al. proposed a ResNet CNN for the identification and classification of various diseases in chili leaves, achieving an overall accuracy of 86.1% (Chaitanya et al., 2023). Chen et al. leveraged the HSV color space for preprocessing chili images, facilitating convolutional neural networks to extract additional features from limited samples of chili leaf disease images. The overall accuracy reached 63.26%, representing a notable improvement of 11.78% compared to traditional RGB (Chen et al., 2023). In 2023, Dai et al. introduced an enhanced lightweight model, GoogLeNet-EL, based on the GoogLeNet architecture for the identification and classification of six types of chili diseases, achieving an overall accuracy of 97.87% (Dai et al., 2023).

Despite significant advancements in deep learning models for chili disease recognition and classification, the pathological symptoms of chili leaf diseases are highly similar, with small inter-class differences, posing challenges to the accuracy of many network models (Wu et al., 2020). Furthermore, acquiring high-quality, diverse datasets of chili leaf disease images still requires considerable effort and time. Additionally, compared to classification models for diseases in other crops, the variety of classification models for chili leaf diseases is relatively limited. Finally, the practical application of chili leaf disease classification models is currently somewhat constrained. Farmers and agricultural practitioners may have insufficient understanding and acceptance of deep learning technologies; they may be more accustomed to traditional agricultural methods and tools. Moreover, employing deep learning models for leaf disease classification may require substantial investment in hardware equipment, data collection, model training, etc. For some small-scale or resource-limited farms, this could pose a significant challenge. To overcome the challenges faced in chili leaf disease classification, this study’s contributions primarily lie in the following aspects:

1. Data aspect: In order to meet the demand for high-quality, diverse training data for deep learning, we performed various image preprocessing operations on the downloaded 500 publicly available chili leaf disease images, including random resizing, random aspect ratio cropping, etc. Additionally, this study introduced data augmentation techniques such as random Gaussian noise, random brightness, random rotation angles, and random occlusion to augment the data to 2500 images. Finally, we divided the dataset into training, validation, and test sets in a ratio of 7:1.5:1.5, providing a sufficient data augmentation solution for the small dataset experiments.
2. Model aspect: To address the issue of singular convolutional kernel size observed in most networks and enhance the model’s ability to learn features from images of different scales, we introduced the Multi-Scale Feature Fusion Module (MSFFM). By adjusting the network block structure, activation functions, and normalization operations, we optimized the overall model. Additionally, this paper introduced the Mixed Channel Spatial Attention Mechanism (MCSAM). Unlike the conventional Channel Spatial Attention Mechanism (CBAM), MCSAM enhances the correlation in non-local regions, improving the model’s capability to extract crucial features.
3. Application aspect: To address the constraints faced in practical applications of chili disease classification, this study developed a chili leaf disease recognition website using the Flask framework. By pre-training the model and deploying it on the web, users can easily identify chili leaf diseases by simply uploading relevant images to the website. This approach allows users to utilize the system without needing to understand deep learning techniques, thus reducing manual labor and associated costs.

The remainder of the paper is organized as follows. Section 2 provides a detailed overview of the overall model architecture and the specific methods employed. Section 3 presents accurate experimental results through a comprehensive comparison of experiments. Section 4 analyzes all research findings, proposes possible improvements, and suggests future research directions. Finally, Section 5 summarizes the main contributions of this study.

## 2 Materials and methods

### 2.1 Datasets

**Figure 1:**
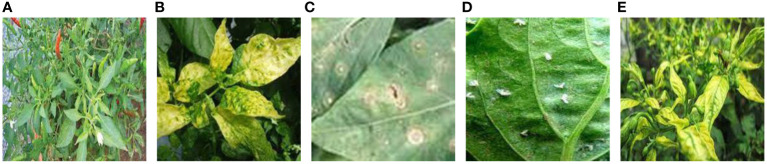
Chili samples. **(A)** Healthy chili leaves. **(B)** Leaf curl disease. **(C)** Leaf spot disease. **(D)** Whitefly disease. **(E)** Yellowing disease. The sample types of 5 kinds of chili leaf disease datasets publicly downloaded from the Internet.

The chili leaf disease dataset is sourced from publicly available datasets on the internet(https://www.kaggle.com/datasets/dhenyd/chili-plant-disease/). The initial dataset consists of 500 images, encompassing five categories: healthy chili leaves, leaf curl disease, leaf spot disease, whitefly, and yellowing disease. Samples of each category are illustrated in **Figure 1**.

**Figure 2:**
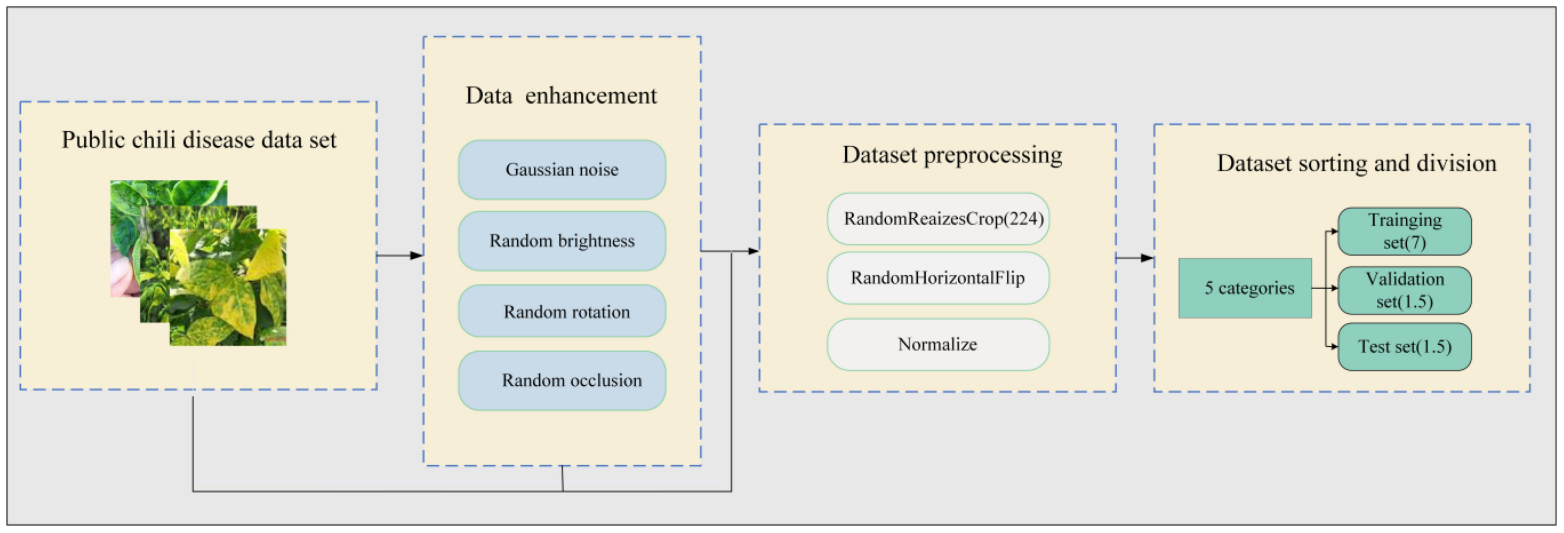
Standardization processing of the chili disease dataset. The original chili leaf disease data set is first enhanced by four kinds of data, such as Gaussian noise, and then preprocessed by three kinds of data, such as clipping, and finally divided into training set, verification set and test set according to proportion.

As shown in **Figure 2**, the chili leaf disease dataset was subjected to preprocessing, data augmentation, and sample categorization, following three standardization procedures (Fujita et al., 2016). This processed dataset was then utilized as the primary experimental dataset.

**Figure 3:**
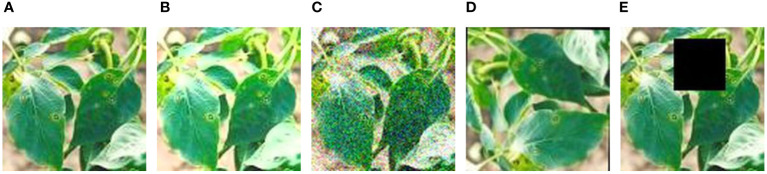
Data augmentation. **(A)** Original. **(B)** Random brightness. **(C)** Random gaussian noise. **(D)** Random rotation angles. **(E)** Random occlusion. Disease samples of chili leaves after four data enhancement methods.

The detailed steps for standardizing the chili leaf disease dataset are as follows:

1. Data Augmentation: Through the introduction of methods such as random Gaussian noise, random brightness adjustments, random rotation angles, and random occlusion, as illustrated in **Figure 3**, we enabled the model to learn more general features, not just adapting to specific samples in the training set. This enhances the model’s robustness. In cases where training data is limited, the model may tend to memorize specific samples and fail to learn general patterns. By introducing these transformations, we expanded the original 500 chili leaf disease images to 2500, increasing their diversity and reducing the risk of overfitting, thus improving the model’s training performance.
2. Image Preprocessing: To comply with the requirements of the convolutional model input, this study employed random cropping with random sizes and aspect ratios, uniformly resizing the original images to a size of 224 × 224, and applying horizontal flipping. To effectively address the issues of gradient vanishing or exploding during model training, we normalized all images in the dataset across the R, G, and B channels to obtain normalized images.
3. Data Set Organization and Division: After undergoing image preprocessing and data augmentation, the original chili leaf disease dataset was randomly divided into training, validation, and test sets according to the typical image split ratio (7:1.5:1.5). **Table 1** illustrates the composition of the final dataset. In this study, the model was trained using the training set, and weights and biases were continuously updated through backpropagation and the Adam optimization algorithm to minimize the loss function on the training set, enabling the model to learn disease features more rapidly. Subsequently, the validation set was employed to assess the disparity between predicted outputs and actual labels. The model’s performance was evaluated after each training epoch to prevent overfitting, further optimizing the model’s parameters. Finally, the performance of the trained model was assessed using the test set to obtain the model’s ultimate accuracy and effectiveness.

**Table 1:** Dataset information.

Categories	Sample number/piece			
	Training sets	Validation sets	Test sets	Total
healthy	350	75	75	500
leaf curl	350	75	75	500
leaf spot	350	75	75	500
whitefly	350	75	75	500
yellowish	350	75	75	500
Total	1750	375	375	2500

### 2.2 The overall design of MCCM model

**Figure 4:**
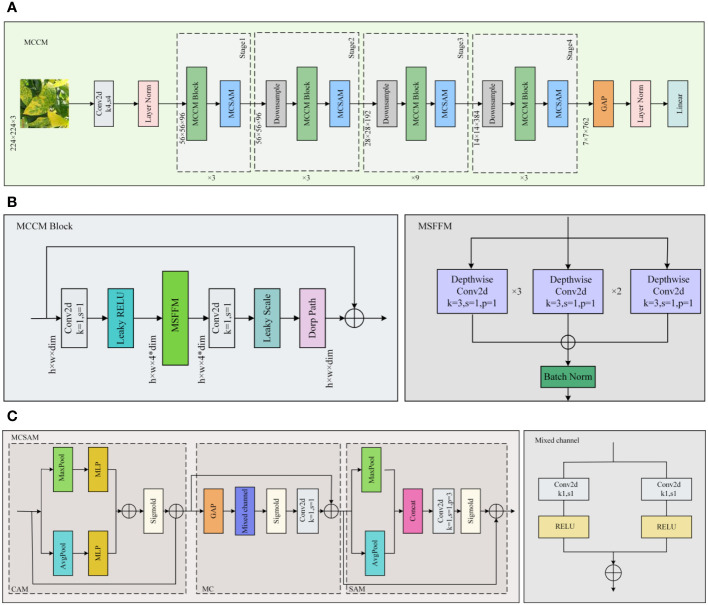
MCCM network model. **(A)** MCCM. **(B)** MCCM Block. **(C)** MCSAM.MCCM model structure is composed of down-sampling, four blocks, and classification layer. The MCCM model consists of MCCM block composed of MSFFM, MCSAM, the down-sampling layer, and the final classification layer.

By enhancing ConvNeXt (Liu et al., 2022), this study introduces the MCCM model, whose overall network architecture is illustrated in **Figure 4**. To validate the rationality and feasibility of the improvement approach, comprehensive comparative experiments were conducted and applied to the recognition and classification of chili leaf diseases. The detailed steps of the improvement method are outlined below:

(1) To enhance the model’s perception of features of various sizes, this study employed a Multi-Scale Feature Fusion Module (MSFFM) composed of depth convolutions with kernel sizes of 3×3, 5×5, and 7×7, replacing the original 7×7 depth convolution in the ConvNeXt module.
(2) By adjusting the Block structure, this study swapped the position of the MSFFM module with a 1×1 convolutional layer. Additionally, we replaced the activation function and normalization operations with appropriate choices. Specifically, GELU was replaced with LReLU, and Layer Norm normalization was replaced with Batch Norm normalization suitable for CNN models. These adjustments were made to further enhance the model’s feature extraction effectiveness.
(3) Improved MCSAM Attention Mechanism, derived from CBAM, was added after each Block layer to increase the sensitivity and effectiveness of the model towards useful features.

### 2.3 Multi-scale feature fusion module

**Figure 5:**
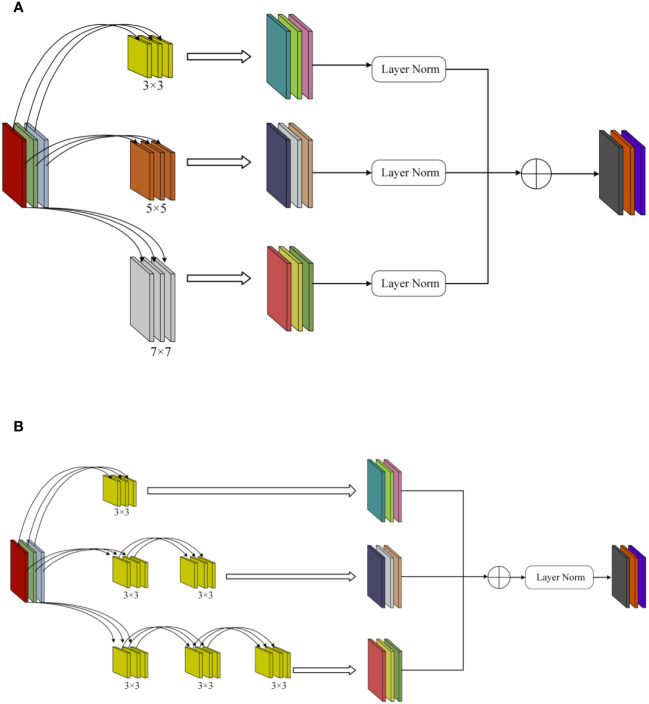
MSFFM structure. **(A)** Before modification. **(B)** After modification. The MSFFM comprises three branches of depth convolution with kernel sizes of 3×3, 5×5, and 7×7. In the depth convolution, multiple 3×3 small convolutional kernels were used to replace the two large convolutional kernels of 5×5 and 7×7, and the LN layer was moved downward.

Due to the presence of various types of diseases in chili leaves, the affected areas vary in size, and their locations on the leaves differ. This results in an uneven distribution of disease features on the leaves. Therefore, in the original Block module of ConvNeXt, using a single 7×7-sized convolutional kernel for depth separable convolution is challenging for effectively extracting detailed disease features of different sizes and those located at the edges of the images. To enhance the model’s sensitivity to features of different sizes and positions, this paper introduced the MSFFM module, as shown in **Figure 5A**.The MSFFM comprises three branches of depth convolution with kernel sizes of 3×3, 5×5, and 7×7. An LN layer is added after each convolutional layer to enhance the stability and effectiveness of the training process model. The utilization of 3×3, 5×5, and 7×7 convolutional kernels enables the network to capture both global and local information within individual modules, facilitating multi-scale feature extraction. This design allows the model to acquire multi-level, multi-scale feature representations that encompass both detailed and holistic information present in the images. Consequently, it aids in better understanding and analyzing the complex structures and features within the images. This is particularly significant for detecting targets of varying sizes, positions, and complexities, enhancing the model’s adaptability and generalization capabilities. Furthermore, the multi-scale feature extraction module exhibits superior robustness when dealing with noise and variations present in images, thereby further improving the model’s robustness and accuracy.

The depth separable convolution, first introduced by MobileNet, is a novel convolutional operation characterized by significantly reducing model parameters and computational workload while only slightly compromising accuracy, leading to a substantial improvement in computational speed (Sandler et al., 2018). In InceptionV2, a method was proposed to replace large convolutions with multiple small convolutions. This strategy effectively reduces the model’s parameter count while maintaining the same feature size, thereby reducing the time required for image inference. In this study, we made adjustments to the original MSFFM module, as illustrated in **Figure 5B**. In the depth convolution, multiple 3×3 small convolutional kernels were used to replace the two large convolutional kernels of 5×5 and 7×7, and the LN layer was moved downward. In the three depth separable convolutional layers, feature fusion was performed first, followed by LN operations. This adjustment not only accelerated the model’s computational speed but also achieved a certain improvement in model recognition accuracy.

### 2.4 Mixed channel spatial attention mechanism

**Figure 6:**
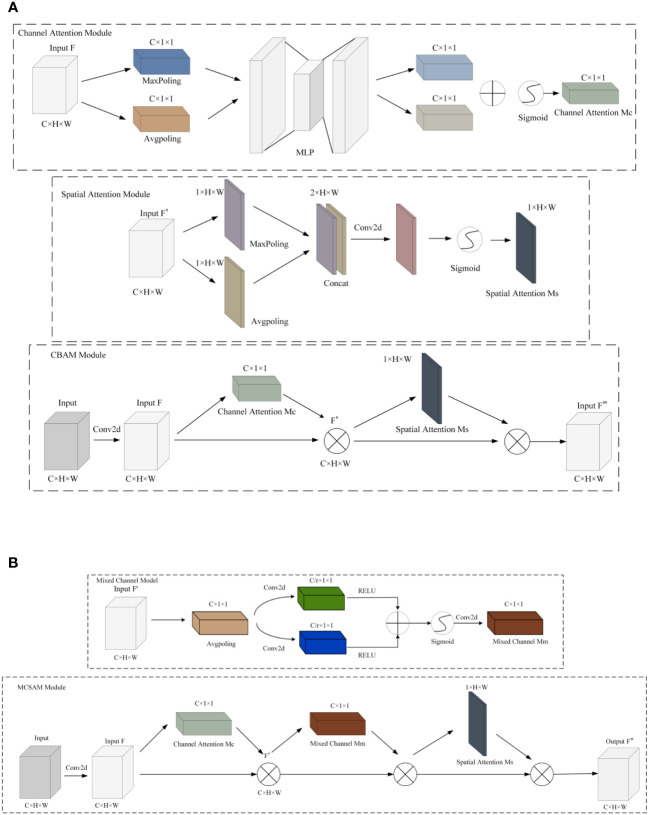
CBAM and MCSAM attention mechanisms. **(A)** CBAM. **(B)** MCSAM. We introduce a hybrid channel attention mechanism between SAM and CAM. This mechanism uses two branch convolution for channel fusion.

Due to its ability to efficiently and accurately determine important features through parameter updates for responsive tasks (Zhang C. J. et al., 2021), attention mechanisms have been widely applied in various domains (Zhang M. et al., 2021; Guo et al., 2022; Wang et al., 2022; Qian et al., 2023). Compared to channel attention mechanisms [such as CA (Li et al., 2019), ECA (Wang et al., 2020), SE (Hu et al., 2018), etc.] and spatial attention mechanisms [such as SimAM (Yang et al., 2021)], the CBAM attention mechanism is a module that combines both channel and spatial attention, proposed by Sanghyun Woo et al. in 2018 (Woo et al., 2018). This module comprises a Channel Attention Module (CAM) and a Spatial Attention Module (SAM). The CAM allows the model to adaptively determine which channels are more crucial for the current task, while the SAM highlights important regions in the image, reducing the impact on model recognition. The structure of this module is depicted in **Figure 6A**.

Although SAM enables the model to establish correlations between regions, its effectiveness is limited to local regions due to the constraints of convolutional operations, restricting connections between different positions within a certain space. To overcome this limitation, this paper introduces a hybrid channel attention mechanism between SAM and CAM. First, the features obtained after CAM operation are globally average-pooled along the channel direction. Next, through two branched convolutions, dimensionality reduction is applied to different channels, and they are fused to form a new feature representation. Subsequently, the features are restored to the original scale through upsampling convolution and multiplied with the input to enhance feature connections between non-local regions. Finally, SAM technology is utilized to enhance correlations between local regions. This improvement in the CBAM attention mechanism overcomes the constraints between local regions and enhances connections between channels in non-adjacent areas. The overall structure is depicted in **Figure 6B**.

### 2.5 MCCM module

According to the original ConvNeXt paper, the authors found similarities between the Inverted Bottleneck module in MobileNetV2 and the MLP module in the Transformer block. Both modules performed well and shared certain similarities in channel design. Therefore, the authors decided to adopt the Inverted Bottleneck as the primary block in the ConvNeXt network, expecting an improvement in model accuracy. However, when attempting to mimic the Transformer model structure by moving the Depthwise Conv layer, originally located in the Block structure, to before the module, it unexpectedly resulted in a decrease in model accuracy. Subsequently, the authors conducted a series of experiments inspired by the Swin Transformer network structure. They changed the convolutional kernel size of the Depthwise Conv from the original 3×3 to 7×7. Additionally, the authors experimented with other kernel sizes, including 3×3, 5×5, 9×9, and 11×11, observing their impact on model accuracy. The experimental results showed that as the kernel size increased, the accuracy exhibited a gradually rising trend. When the kernel size reached 7×7, the accuracy reached a saturation point, remaining unchanged compared to the original 3×3 kernel size before the upward movement. This indicates that the model with the Depthwise Conv layer moved upward and a kernel size set to 7×7 achieved a level of accuracy equivalent to the model with the original 3×3 kernel size before the upward movement.

Therefore, to further optimize the ConvNeXt module, this study decided to move the existing Depthwise Conv layer downward in the module and experiment with different kernel sizes (3×3, 5×5, 7×7, and 9×9). Surprisingly, experimental results indicated that placing the Depthwise Conv layer in the middle of the module and using a 3×3-sized kernel was sufficient to maintain the original accuracy. As the kernel size increased, the accuracy showed an upward trend, reaching a saturation point when the kernel size reached 7×7. Further investigation revealed that using a single kernel size (3×3, 5×5, 7×7) resulted in a continuous improvement in model accuracy. Inspired by this, this study considered improving the model by using a multi-scale fusion module (MSFFM) composed of kernels of sizes 3×3, 5×5, and 7×7 to enhance the model’s sensitivity to features of different sizes. Experimental results showed a significant improvement in model accuracy after this modification.

### 2.6 LRELU activation function and batch norm

**Figure 7:**
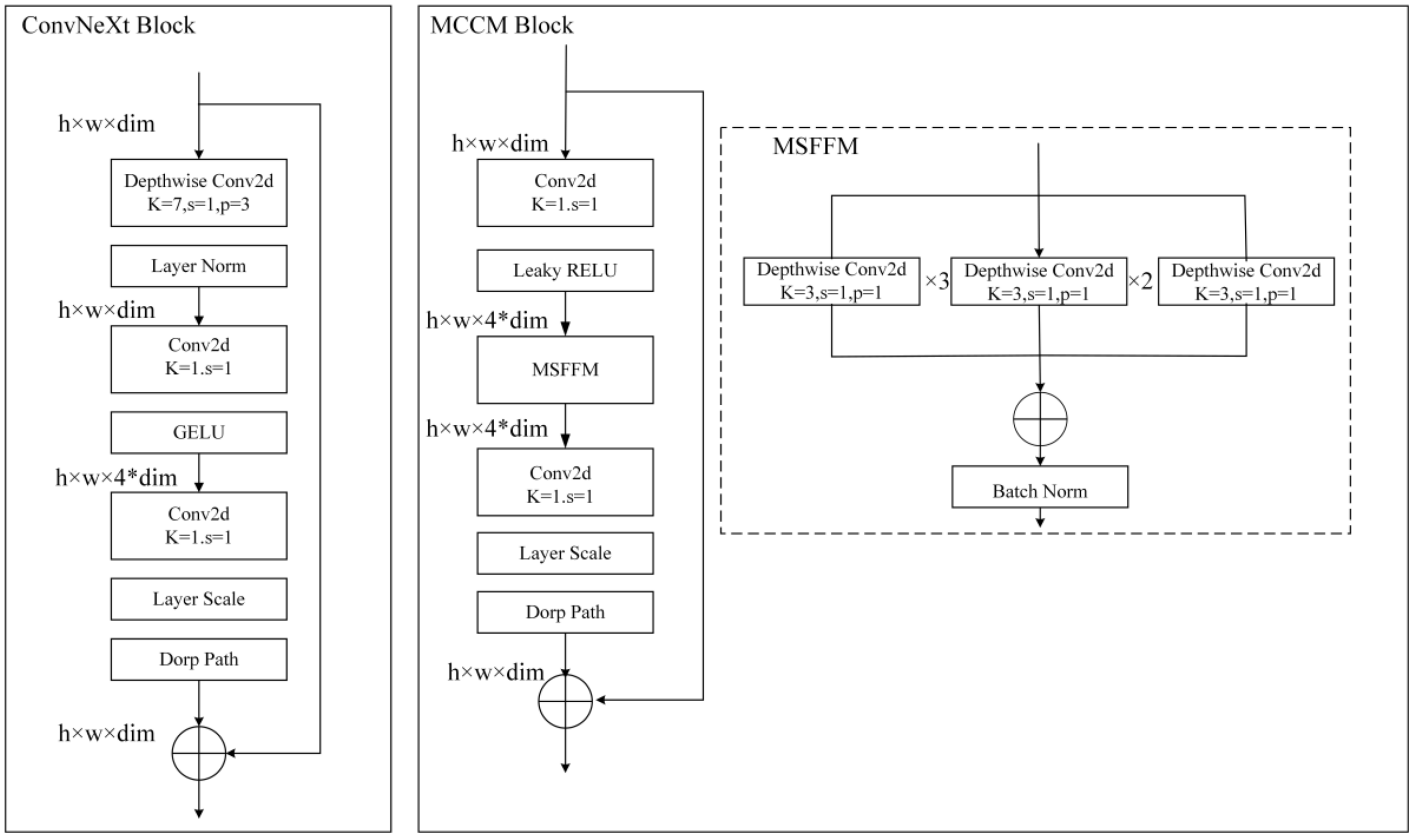
ConvNeXt block and MCCM Block. Compared to the original ConvNeXt block, in MCCM block, the MSFFM layer is moved down and GELU and LN are replaced with LRELU and BN.

As shown in **Figure 7**, for further optimization of the improved module, this study implemented two crucial adjustments: firstly, replacing the GELU activation function in the original block with LRELU; secondly, replacing the originally used LN operation with BN. Specifically, in the MSFFM module, the previously shared LN was substituted with BN.

#### 2.6.1 LRELU and GELU

In the process of emulating the Transformer, ConvNeXt decided to replace the common RELU activation function in convolutional neural networks with the widely used GELU activation function in the Transformer (Hendrycks and Gimpel, 2016). However, surprisingly, despite this change, the model’s accuracy did not show any improvement. As shown in **Supplementary Figure S1A**, the GELU used in the original ConvNeXt block, compared to some other activation functions like RELU (Glorot et al., 2011), has a well-defined and continuous derivative across the entire real number range. Its mathematical expression is given by Equation 1:


(1) $$GELU\left(\text{x}\right)=0.5\left(\sqrt{\frac{2}{\text{π}}}\left(\text{x}\left(1+\text{tanh}x+0.044715{\text{x}}^{3}\right)\right)\right)$$

Where, tanh is the hyperbolic tangent function, $\sqrt{\frac{2}{\pi }}$ is a constant.

Although GELU lacks an upper bound, making it less susceptible to gradient saturation, and has a lower bound, providing stronger regularization effects, along with its smoothness aiding in improving optimization algorithm performance, it may introduce numerical instability in certain cases. This is especially true when the input is predominantly negative and exceeds the lower bound. Such instability can lead to issues like vanishing or exploding gradients, particularly in deep neural networks. To address these limitations, this experiment opts to use the LRELU activation function as a replacement for GELU. According to the mathematical expression of LRELU (Equation 2) and its derivative (Equation 3), LRELU maintains a non-zero gradient for negative inputs, thus avoiding neuron deactivation (Xu et al., 2015). Additionally, with its adjustable negative slope, LRELU can learn more complex patterns.


(2) $$\text{f}\left(\text{x}\right)=\{\begin{array}{c} \text{x},\;\text{x}\geq 0\\ \text{αx},\text{x}<0 \end{array}\;\text{α}\in \left(0,1\right)$$

(3) $${\text{f}}^{\prime }\left(\text{x}\right)=\{\begin{array}{c} 1,\;\text{x}\geq 0\\ \text{α},\text{x}<0 \end{array}\;\alpha \in \left(0,1\right)$$

Therefore, we replaced the GELU activation function with LRELU in the original ConvNeXt block, as illustrated in **Supplementary Figure S1B**. LRELU exhibits a slight slope on negative input values rather than being zero, thereby enhancing the model’s nonlinearity and performance. Additionally, LRELU reduces the likelihood of overfitting by introducing noise during the training process and increasing the model’s randomness. Compared to other activation functions, LRELU has advantages such as high computational efficiency, robustness, and better generalization than GELU. Furthermore, LRELU can prevent the issue of dead neurons and contribute to faster model convergence.

#### 2.6.2 Batch norm and layer norm

To enhance the stability of the neural network, the input data is initially normalized using Equation 4 before being passed to the neurons. This normalization process ensures that the input data conforms to a standard distribution within a fixed range.


(4) $$\text{h}=\text{f}\left(\text{g}\cdot \frac{\text{x}-\text{μ}}{\text{σ}}+\text{b}\right)$$

Where μ  is translation parameter, σ  is scaling parameter, b  is re-translation parameter, g is re-scaling parameter, and the obtained data conforms to the distribution of mean b and variance g square.

According to the different dimensions of normalization operations, it can be distinguished between BN (Batch Normalization) and LN (Layer Normalization). BN normalizes each feature within a batch (Ioffe and Szegedy, 2015). According to the calculation Equation 5 of BN, this method targets individual neurons by computing the mean and variance of the neuron ${\text{x}}_{\text{i}}$ using a small batch of data during network training.


(5) $$\mu_{i}=\frac{1}{\text{M}}\sum X_{i},\;\sigma_{i}=\sqrt{\frac{1}{\text{M}}\sum {\left(X_{i}-\mu_{i}\right)}^{2}+\text{ε}}$$

Where M is the minimum batch size.

LN normalizes the distribution of a layer by normalizing along the Hidden size dimension (Ba et al., 2016). According to the calculation Equation 6 of LN, which takes into account all dimensions of input in a layer, it computes the mean input value and input variance for the layer. Then, it employs the same normalization operation to transform the input along each dimension.


(6) $$\text{μ}=\sum_{\text{i}}X_{i},\;\text{σ}=\sqrt{\sum_{\text{i}}{\left(X_{i}-\text{μ}\right)}^{2}+\text{ε}}$$

Where i represents all the input neurons in the layer. The two parameters μ ,  σ in the standard formula for the transformation are all scalars (as opposed to vectors in BN), indicating that all inputs share the same normalized transformation.

In contrast to LN, BN introduces additional noise on each small batch of data, thereby reducing overfitting (sometimes serving as a regularization mechanism). In contrast, LN normalizes over the features of each individual sample, thus lacking batch-level noise. BN standardizes the inputs for each layer, ensuring that each layer receives inputs with similar distributions, accelerating the training process of neural networks, alleviating gradient vanishing and exploding issues, and making gradient propagation more straightforward. While LN also provides some benefits in terms of gradient propagation, generally, BN tends to achieve faster convergence in large deep networks. Ultimately, BN often performs well in deep feedforward networks, whereas LN is typically more effective when dealing with recurrent neural networks and sequence models. Experimental results indicate that replacing LN with BN leads to improved model accuracy. This suggests that in the MCCM model, Batch Normalization is more suitable compared to Layer Normalization.

### 2.7 Chili leaf disease classification system design

**Figure 8:**
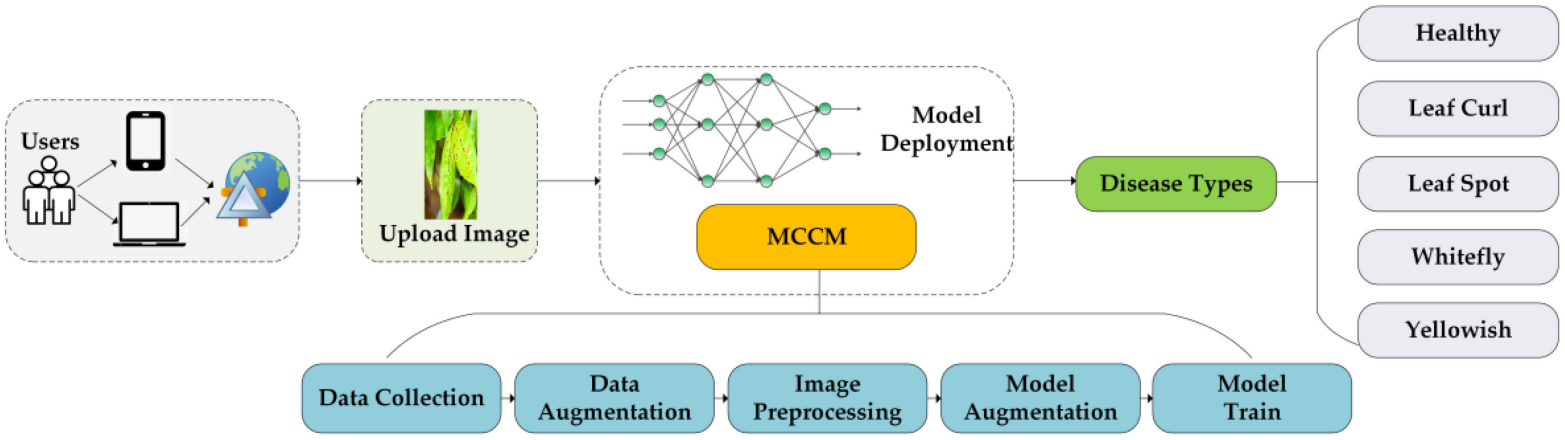
Chili leaf disease classification system. Users use mobile devices to upload chili leaf disease pictures to the web page, and identify the disease through the trained MCCM model.

Despite existing research on the classification of chili leaf diseases, the practical application of this field remains relatively limited. In this study, we have successfully developed a web-based application for the classification of chili leaf diseases, as illustrated in **Figure 8**. Users can upload images of chili leaf diseases, and the system will automatically recognize and classify them into different disease types. The application is built on the Flask framework, allowing users to access it through a web browser. The backend of the application utilizes trained MCCM model weights to generate the corresponding classification results.

## 3 Experimental results and analysis

### 3.1 Experimental environment

The experimental setup utilized version 1.11 of the Pytorch deep learning framework, programmed in Python 3.8. The experiments were conducted on an Intel(R) Xeon(R) Platinum 8352V CPU and an NVIDIA RTX4090 GPU. To ensure the validity of the experimental results, each model was configured with identical hyperparameters, including a fixed number of epochs (100) and batch size (32).

### 3.2 Model evaluation

During this study, we obtained the optimal weights for each model through training and subsequently conducted testing and analysis on a designated test set. In the context of chili leaf disease classification research, commonly used model evaluation metrics include accuracy, recall, precision, and F1 score. Additionally, the application of a confusion matrix allows for a more detailed analysis of the model’s classification performance and misclassifications. In this study, the four elements of the confusion matrix are defined as True Positive (TP), False Positive (FP), False Negative (FN), and True Negative (TN).

In model evaluation, accuracy refers to the proportion of correctly classified samples to the total number of samples. In multi-class problems, macro-averaged precision and micro-averaged precision are commonly used for a comprehensive evaluation of performance. Accuracy provides an understanding of the overall performance of the model in the classification task. Precision, also known as positive predictive value, quantifies the proportion of true positive samples recognized by the model among all positive samples. High precision indicates more accurate identification of positive samples by the model, minimizing false positive predictions and reducing unnecessary operations. Recall is a metric that measures the proportion of true positive samples identified by the model from all true samples. High recall indicates the model’s ability to accurately identify true positive samples, demonstrating good discriminatory power. Recall helps understand the model’s ability to capture all instances of the disease, ensuring that no potential cases are missed. The F1 score is a balanced metric that comprehensively evaluates the balance between model accuracy and recall. A higher F1 score indicates a better balance between precision and recall. The F1 score ensures that the model performs well in both accurately identifying the disease and capturing all instances. These metrics are represented by Equations 7–10 as shown below:


(7) $$Accuracy=\frac{\left(TP+TN\right)}{\left(TP+TN+FP+FN\right)}$$

(8) $$Precision=\frac{TP}{\left(TP+FP\right)}$$

(9) $$Recall=\frac{TP}{\left(TP+FN\right)}$$

(10) $$\text{F}1-Score=\frac{2\times \left(Precision\times Recall\right)}{\left(Precision+Recall\right)}$$

### 3.3 Comparison experiment before and after modification of MSFFM module

To validate the effectiveness of replacing the 7×7 depthwise separable convolution kernel in the original Block module of the ConvNeXt model with the MSFFM (Multi-Scale Feature Fusion Module), experiments were conducted using a preprocessed dataset of chili diseases. The experiments were carried out on the original ConvNeXt model, the model after adding the original MSFFM, and the model with a shared Layer Norm normalization in the MSFFM. The validation was performed by comparing accuracy and loss values in the experimental results. The experimental results on the chili disease dataset are presented in **Table 2**. The original ConvNeXt block achieved an accuracy of 86.3%. When replaced with the MSFFM module, the accuracy increased by 1.2%, and the loss decreased by 0.075. For the MSFFM model with shared Layer Norm normalization, although the loss slightly increased, the accuracy reached 88.4%, which is an improvement of 2.1% compared to the original ConvNeXt block and 0.9% compared to the original MSFFM model. These experiments demonstrate the effectiveness of the MSFFM module in enhancing network feature extraction.

**Table 2:** Comparison of experimental results before and after modification of MSFFM module.

Test ID	Depthwise conv	Accuracy (%)	Loss
1	7×7, s=1, p=3	86.3	0.374
2	Primary MSFFM	87.5	0.299
3	MSFFM	88.4	0.316

### 3.4 Comparison experiment before and after MCCM module optimization

To validate the effectiveness of the MCCM module after optimizing the ConvNeXt module, this study conducted tests on a dataset of chili leaf diseases. We utilized different kernel sizes (3×3, 5×5, 7×7, and 9×9) for the downward shifted Depthwise Conv layer and introduced the MSFFM module for comparative experiments. Additionally, we focused on improving the accuracy of the proposed model by adjusting different activation functions and normalization techniques. Based on the modified structure of the proposed MCCM model, this paper made adjustments to both activation functions and normalization, replacing the GELU activation function with LRELU and the original LN operation with BN. The results of the comparative experiments before and after optimizing the MCCM module are presented in **Table 3**.

**Table 3:** Module optimization experiment results.

Test ID	First Layer	AF/Norm	Second Layer	AF/Norm	Accuracy (%)	Loss
1	7×7, s1, p3	LN	1×1, s1, p0	GELU	86.3	0.374
2	MSFFM	LN	1×1, s1, p0	GELU	88.4	0.316
3	1×1, s1, p0	GELU	3×3, s1, p1	LN	86.3	0.394
4	1×1, s1, p0	GELU	5×5, s1, p2	LN	86.7	0.370
5	1×1, s1, p0	GELU	7×7, s1, p3	LN	87.0	0.350
6	1×1, s1, p0	GELU	9×9, s1, p4	LN	86.8	0.363
7	1×1, s1, p0	GELU	MSFFM	LN	88.1	0.324
8	1×1, s1, p0	LRELU	MSFFM	LN	88.5	0.310
9	1×1, s1, p0	LRELU	MSFFM	BN	89.6	0.271

By comparing the experimental results, it was observed that shifting the Depthwise Conv layer to the middle of the block, using a 3×3 kernel, could maintain the original accuracy of 86.3%. As the kernel size increased (3×3, 5×5, 7×7), the accuracy slightly improved from 86.3% to 87%. However, when the kernel size reached 9×9, there was a slight decrease in model accuracy, indicating that a 7×7 kernel size reached saturation. Furthermore, after replacing with the MSFFM module, the accuracy improved again to 88.1%. This experimental result not only validates the effectiveness of shifting the Depthwise Conv layer but also confirms that the multi-scale fusion module (composed of 3×3, 5×5, 7×7 kernel sizes) enhances the model’s sensitivity to features of different sizes.

The experimental results once again confirm that replacing the GELU activation function in the original block with LRELU increases accuracy by 0.4%. Compared to the LN used in the original block, using BN increases the model’s accuracy by 1.1%, reaching 89.6%, and the loss value is 0.271, the lowest among all experimental models. This indicates that the adjustment of activation functions and normalization operations brings overall optimization to the model, validating the effectiveness of these adjustments.

### 3.5 Performance comparison of different attention modules

To enhance the connection between input features across channels, non-local regions, and spatial dimensions, this study introduced the MCSAM attention mechanism module. To verify the superior performance of MCSAM compared to other attention mechanisms, this paper conducted comparative experiments by adding CA, ECA, SE, SimAM, and CBAM attention mechanisms to the blocks at the same positions. The experimental results are shown in **Table 4**. The study indicates that, except for a slight decrease in model accuracy after adding the SimAM attention mechanism, other attention mechanisms improve the model’s performance. From the experimental data, the added MCSAM attention mechanism performs the best, with an accuracy of 91.2%, an improvement of 1.6% compared to the original model. Additionally, the loss value of the MCSAM module is the lowest among all experimental models, at 0.236, a decrease of 0.035 compared to the original model. Therefore, compared to other attention mechanisms, the MCSAM attention module demonstrates outstanding performance in model accuracy.

**Table 4:** The impact of different attention modules on classification results.

Test ID	Attention Module	Accuracy (%)	Loss
1	Original Block	89.6	0.271
2	+CA	90.0	0.225
3	+ECA	90.4	0.273
4	+SimAM	88.3	0.321
5	+CBAM	90.6	0.257
6	+MCSAM	91.2	0.236

### 3.6 Ablation experiment

To validate the improvement brought by the optimized MSFFM module and MCSAM attention mechanism, ablation experiments were conducted by integrating the MSFFM module and MCSAM into the ConvNeXt network. The results are shown in **Table 5**.

**Table 5:** Experimental results of ablation of different modules.

Model	Factors		Accuracy(%)	Loss
	MSFFM	MCSAM		
ConvNeXt	×	×	86.3	0.374
ConvNeXt-MSFFM	√	×	89.6	0.271
ConvNeXt-MCSAM	×	√	88.3	0.314
MCCM	√	√	91.2	0.236

According to the analysis results in **Table 5**, integrating the optimized MSFFM module into the ConvNeXt network not only improved the accuracy by 3.3% but also resulted in a decrease in loss. The results of disease classification testing show that compared to the ConvNeXt model, the ConvNeXt-MSFFM model exhibits improvements in identifying healthy chili leaves and notably enhances the feature extraction of leaf curl. Additionally, there is a slight improvement in the detection accuracy of leaf spot and yellowing diseases. This indicates that the module contributes to enhancing the model’s ability to extract features of different sizes and dimensions to some extent. Additionally, incorporating the MCSAM attention mechanism into the ConvNeXt network led to a 2% increase in accuracy and a decrease in loss. The test results also indicate significant improvements in the ConvNeXt-MCSAM model’s recognition of healthy chili leaves, leaf curl, and yellowing diseases. This suggests that adding the MCSAM attention mechanism effectively strengthens the feature connections between non-local regions, thereby enhancing the model’s capability to extract crucial features, consequently improving accuracy and reducing loss values. Finally, compared to the ConvNeXt, ConvNeXt-MSFFM, and ConvNeXt-MCSAM models, the MCCM model performs better on the five types of chili leaf diseases. Although there was no significant improvement in detecting yellowing diseases, there was a significant enhancement in the recognition capability of other diseases, further confirming the superiority of the MCCM model.

### 3.7 Utilization of transfer learning

To expedite training and enhance prediction accuracy, we employed transfer learning by pretraining the proposed MCCM model on a large-scale image classification task with a vast dataset. Subsequently, we utilized the pretrained weights obtained from this task and applied them to our specific target task. This approach leverages existing knowledge, obviating the need to train the model from scratch, thereby improving efficiency and performance. Therefore, this paper pretrained the MCCM model using the publicly available Plant Village dataset and applied the obtained pretrained weights to the focal research task of classifying diseases in chili leaf images. To preserve the high-level feature representations acquired through pre-training, prevent excessive adjustments of these features on the chili leaf disease classification task, enhance model generalization, and reduce the risk of overfitting, the model’s corresponding classification layer was frozen during the transfer learning process.

**Figure 9:**
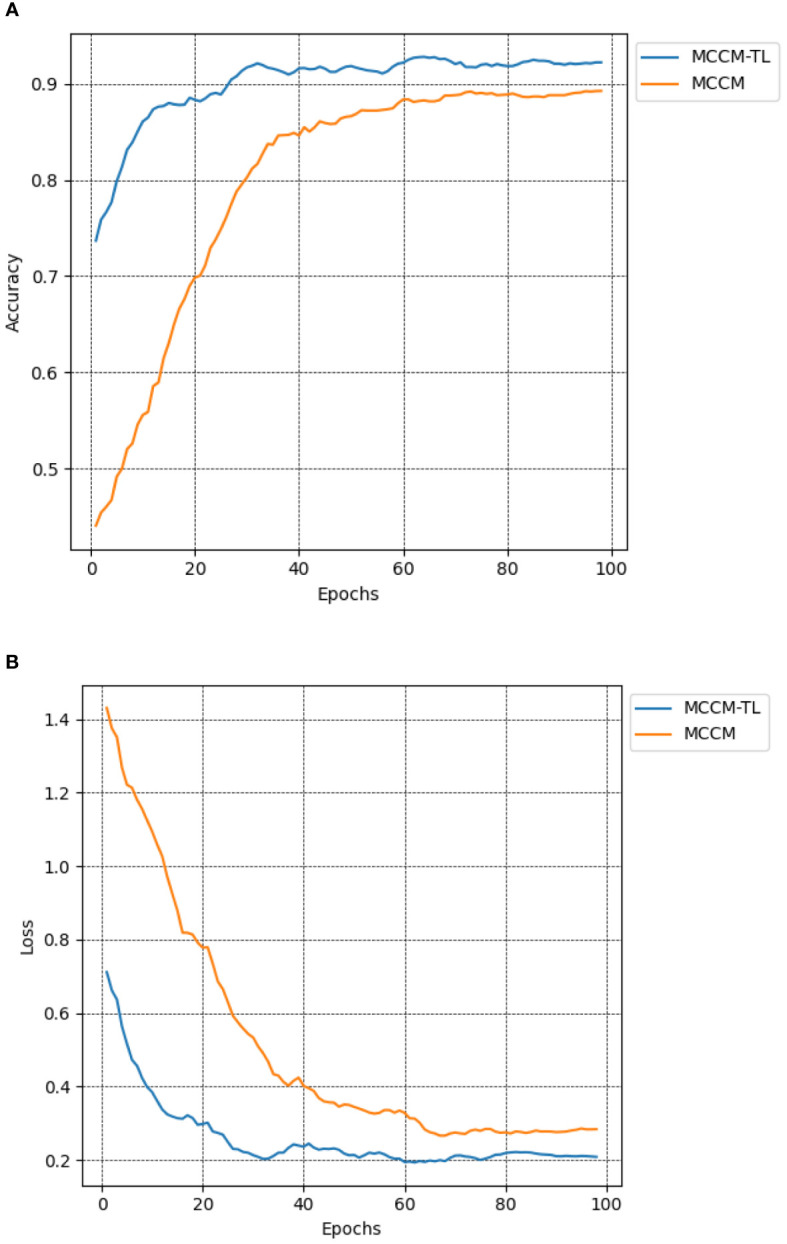
Comparison of accuracy and loss of MCCM model without transfer learning and transfer learning. **(A)** Accuracy. **(B)** Loss. Accuracy curve and loss curve of MCCM model under transfer learning and MCCM model without transfer learning under the verification set of chili leaf disease and epoch 100. The horizontal coordinate is the number of epochs, and the vertical coordinate is the corresponding accuracy and loss value.

To investigate the impact of transfer learning on classification results, this paper compared the MCCM model with and without transfer learning in terms of recognition accuracy on the chili leaf disease test set and the loss value on the validation set. The experimental results shown in **Figure 9**; **Table 6** indicate that the MCCM model, when applied with transfer learning, achieves an accuracy of 93.5% and a loss value of 0.192. Compared to the scenario without transfer learning, the accuracy improves by 2.3%, and the loss value decreases by 0.044. Therefore, the application of transfer learning not only accelerates the convergence speed of the model but also significantly improves its accuracy.

**Table 6:** The impact of transfer learning on classification results.

Test ID	Transfer Learning	Accuracy (%)	Loss
1	Non-Use	91.2	0.236
2	Use	93.5	0.192

### 3.8 Network Grad-CAM visualization

**Figure 10:**
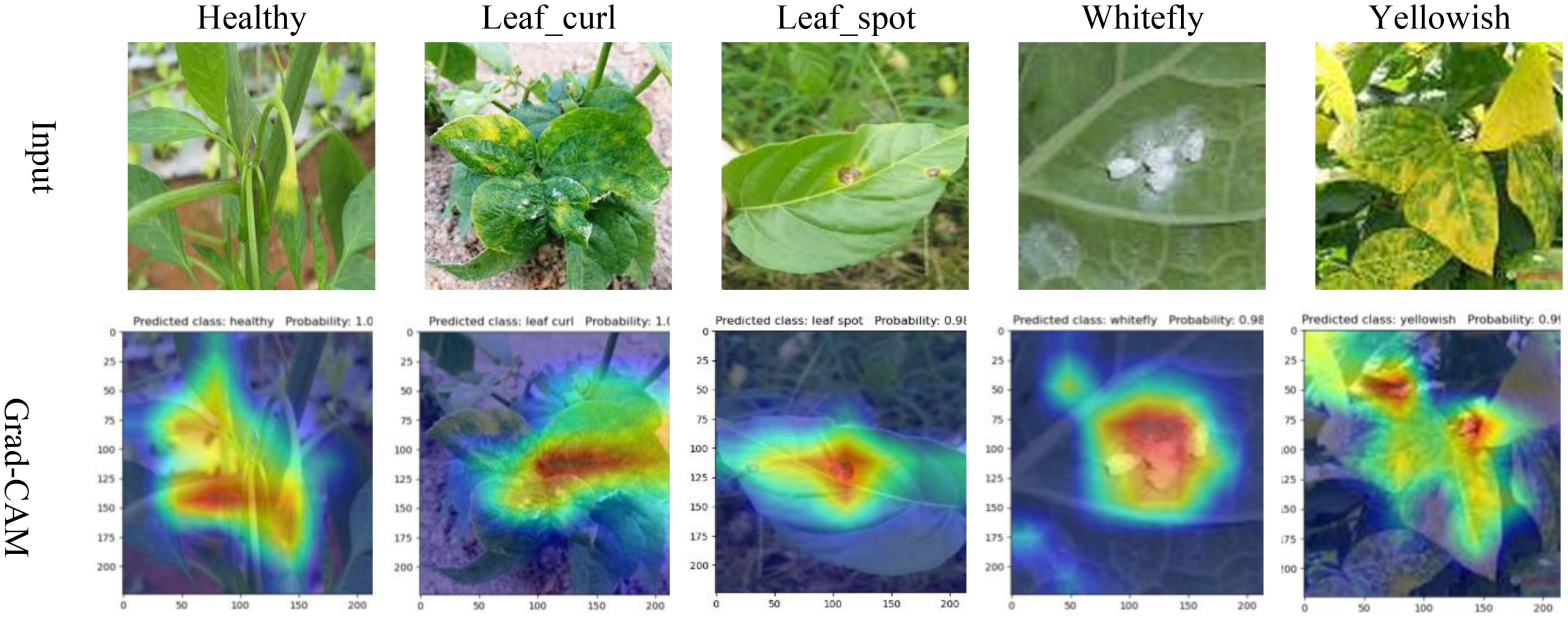
Comparison of Grad-CAM heat maps.

To better observe the learning capability of the MCCM model on chili leaf disease features in this experiment, we predicted partial data of each disease in the test set and visualized them using Grad-CAM. In this study, we selected the last layer of the MCCM model as the network’s feature visualization layer for feature visualization, as shown in **Figure 10**. Through observing the visualization results, we found that the MCCM model not only accurately predicted the classification results for each disease but also accurately identified key areas of different disease categories. Additionally, we noticed that the model paid less attention to irrelevant complex backgrounds such as soil and vegetation surrounding the leaf diseases. Furthermore, the model exhibited high accuracy in identifying small regions of diseases such as yellowing disease and leaf spot disease on the leaves. Therefore, these results validate the strong learning capability of the MCCM model on chili leaf disease features.

### 3.9 Performance comparison of different models

**Figure 11:**
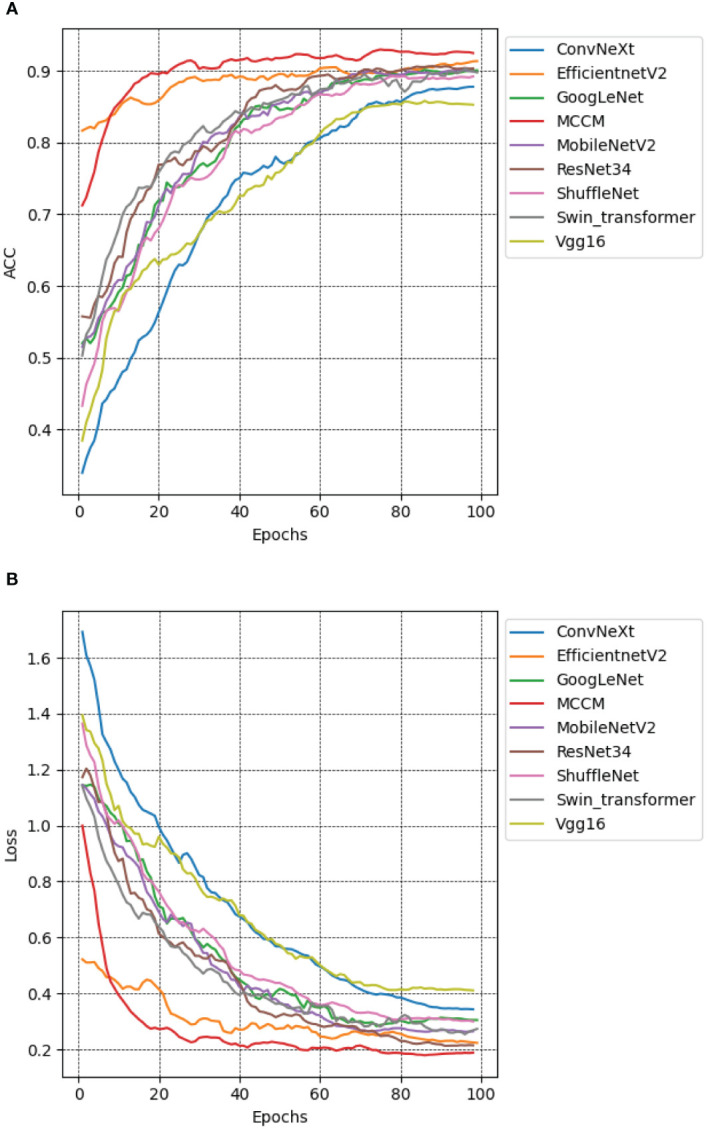
Accuracy and loss performance of MCCM model and other models. **(A)** Accuracy. **(B)** Loss. Different color curves represent the accuracy and loss values of different models on the verification set of chili leaf disease and under epoch 100. The horizontal coordinate is the number of epochs, and the vertical coordinate is the accuracy and loss value corresponding to each model.

To evaluate the performance of the MCCM model, this study employed the chili leaf disease dataset and conducted training and testing on various models, including the MCCM model, as well as Vgg16, ResNet34 (He et al., 2016), GoogLeNet, MobileNetV2, ShuffleNet, EfficientNetV2, ConvNeXt, and Swin-Transformer (Liu et al., 2021). By comparing the accuracy, loss, precision, recall, and F1 values of each model, the results are presented in **Figure 11**, as well as **Supplementary Table S1**. The research indicates that the MCCM model exhibits a 1.5% improvement in accuracy compared to the Swin-Transformer model and a 1.1% improvement compared to the ResNet34 model, which is a high-performing CNN convolutional model in terms of accuracy. Notably, the MCCM model achieved the lowest loss value among all models, only 0.192. Furthermore, the model outperforms others significantly in terms of precision, recall, and F1 score. Compared to the EfficientNetV2 model, which performs well in these metrics, the MCCM model shows improvements of 0.82%, 0.8%, and 0.8%, respectively. In summary, the MCCM model demonstrates superior performance compared to other models. In contrast to the original ConvNeXt model, the MCCM model not only achieves a significant improvement in accuracy but also excels in metrics such as loss, precision, recall, and F1 score, with improvements of 7.2%, 5.68%, 5.46%, and 5.55%, respectively.

Additionally, the classification performance of the model was further assessed using a confusion matrix. **Supplementary Figure S2** illustrates the confusion matrix results of this model compared to eight other models. The model demonstrates favorable classification performance across five categories: healthy chili leaves, leaf curl disease, leaf spot disease, whitefly, and yellowing disease. However, due to the potential similarity in appearance between symptoms of leaf curl disease and yellowish disease, such as color changes and abnormal shapes, and compared to the Swin-Transformer model with a stronger self-attention mechanism, the effectiveness of the MCCM model in distinguishing general symptoms of leaf curl disease and yellowish disease may be slightly insufficient. While the model may not exhibit optimal results in all class distinctions, its overall classification performance remains impressive.

### 3.10 MCCM model generalization

#### 3.10.1 K-fold cross-validation

**Figure 12:**
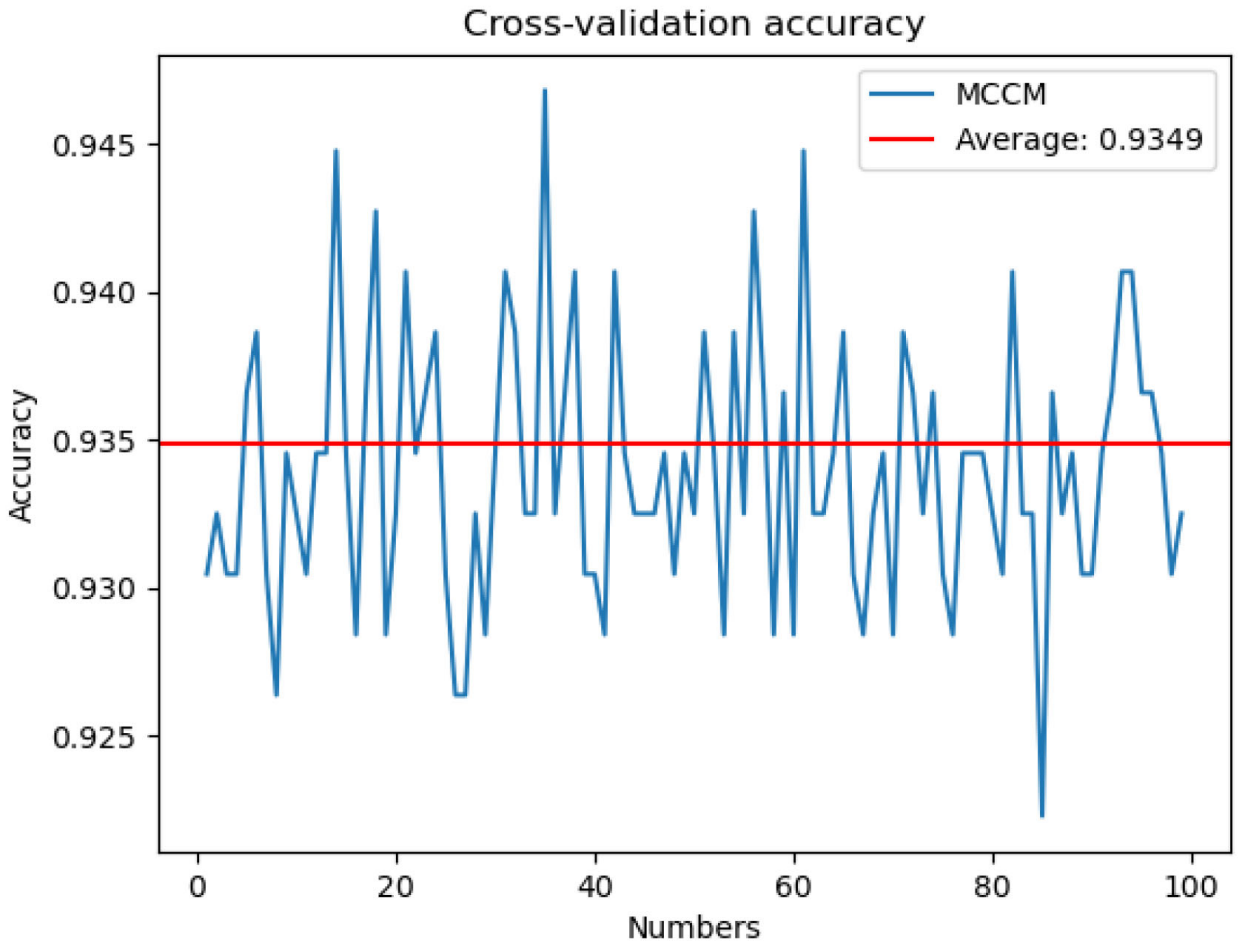
Cross-validation accuracy. The MCCM model was cross-validated 100 numbers on the disease data of chili leaves and the corresponding accuracy rate of each cross-validation.

To address potential biases in evaluation results arising from specific categories or patterns within the image dataset, this study implemented a robust training approach using 100-fold cross-validation for the proposed MCCM model. The dataset was partitioned into 100 subsets, with 99 subsets utilized for training in each iteration, leaving one subset for testing. The model underwent a total of 100 training epochs, each time with a different subset as the test set. The accuracy on the test set, as depicted in **Figure 12**, was recorded for each epoch. The resulting average accuracy across all folds reached 93.49%. This meticulous 100-fold cross-validation strategy was employed to mitigate the impact of randomness and enhance the model’s generalization capabilities, ensuring more reliable and comprehensive performance evaluation.

#### 3.10.2 Rice and maize disease test results

**Figure 13:**
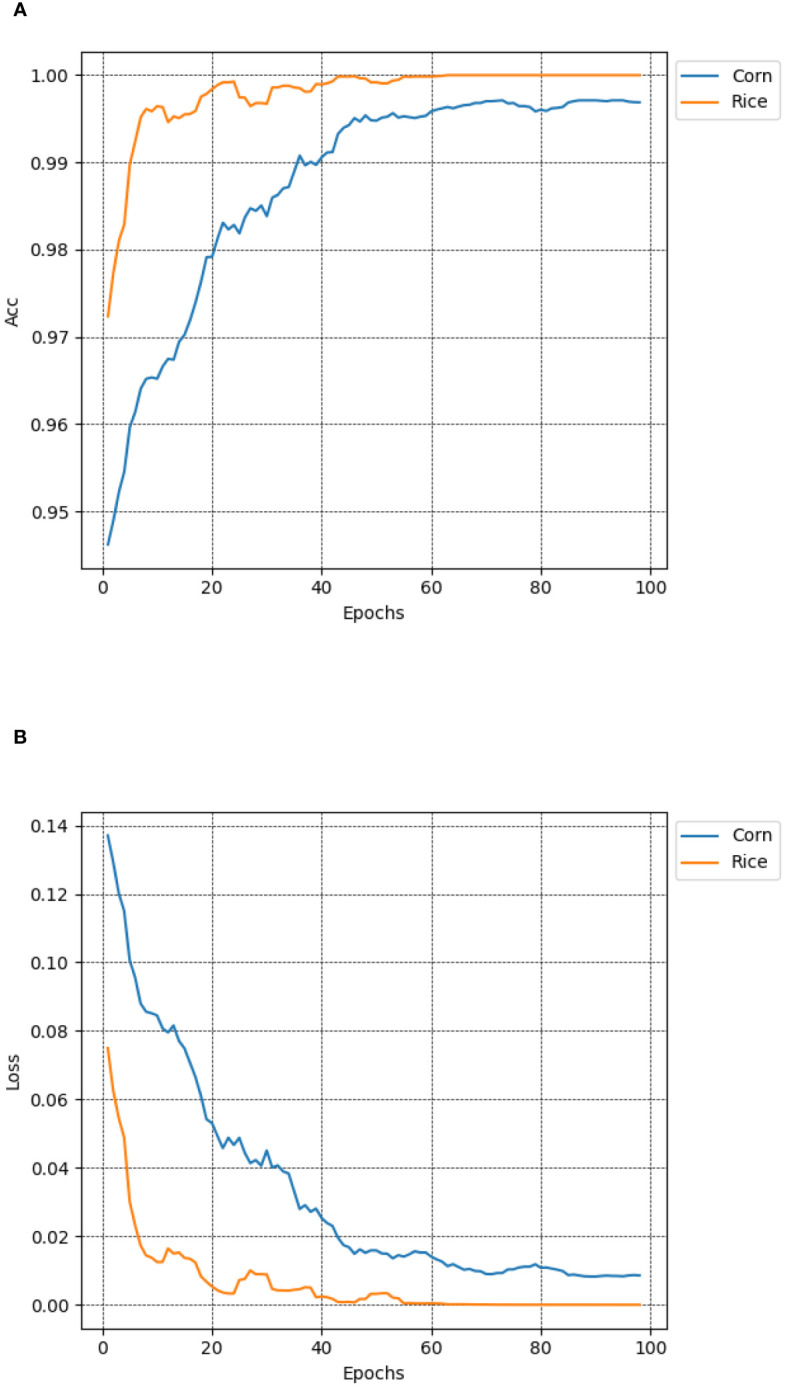
Accuracy and loss of MCCM on maize and rice disease datasets. **(A)** Accuracy. **(B)** Loss. In the figure, the blue curve and the yellow curve represent the accuracy curve and loss curve of the maize and rice leaf disease verification set in the MCCM model and epoch 100, respectively. The horizontal coordinate represents the number of epochs, and the vertical coordinate represents the corresponding accuracy and loss value.

To validate the generalization ability of the MCCM model on different crop diseases, this study conducted performance tests using publicly available datasets, including rice leaf disease dataset and maize leaf disease dataset (https://www.kaggle.com/datasets/nirmalsankalana/rice-leaf-disease-image and https://www.kaggle.com/datasets/smaranjitghose/corn-or-maize-leaf-disease-dataset). The rice leaf disease dataset covers four types: rice blast, bacterial leaf blight, brown spot, and rice tungro disease, while the maize leaf disease dataset includes healthy states and three types of diseases: gray leaf spot, rust, and leaf blight. To ensure dataset diversity, data augmentation techniques such as random Gaussian noise, random brightness, random rotation angles, and random occlusion were employed, along with random cropping, horizontal flipping, and image normalization preprocessing methods. As shown in **Figure 13**, the test results revealed that the MCCM model achieved an impressive accuracy of 99.7% and a loss value of only 0.0096 in maize disease classification. In rice disease classification, the accuracy reached 99.8%, with an exceptionally low loss value of 0.00028. This indicates that the MCCM model exhibits excellent performance in the classification of rice and maize diseases, validating its generalization capability across different crop diseases.

### 3.11 Chili leaf disease classification system

**Figure 14:**
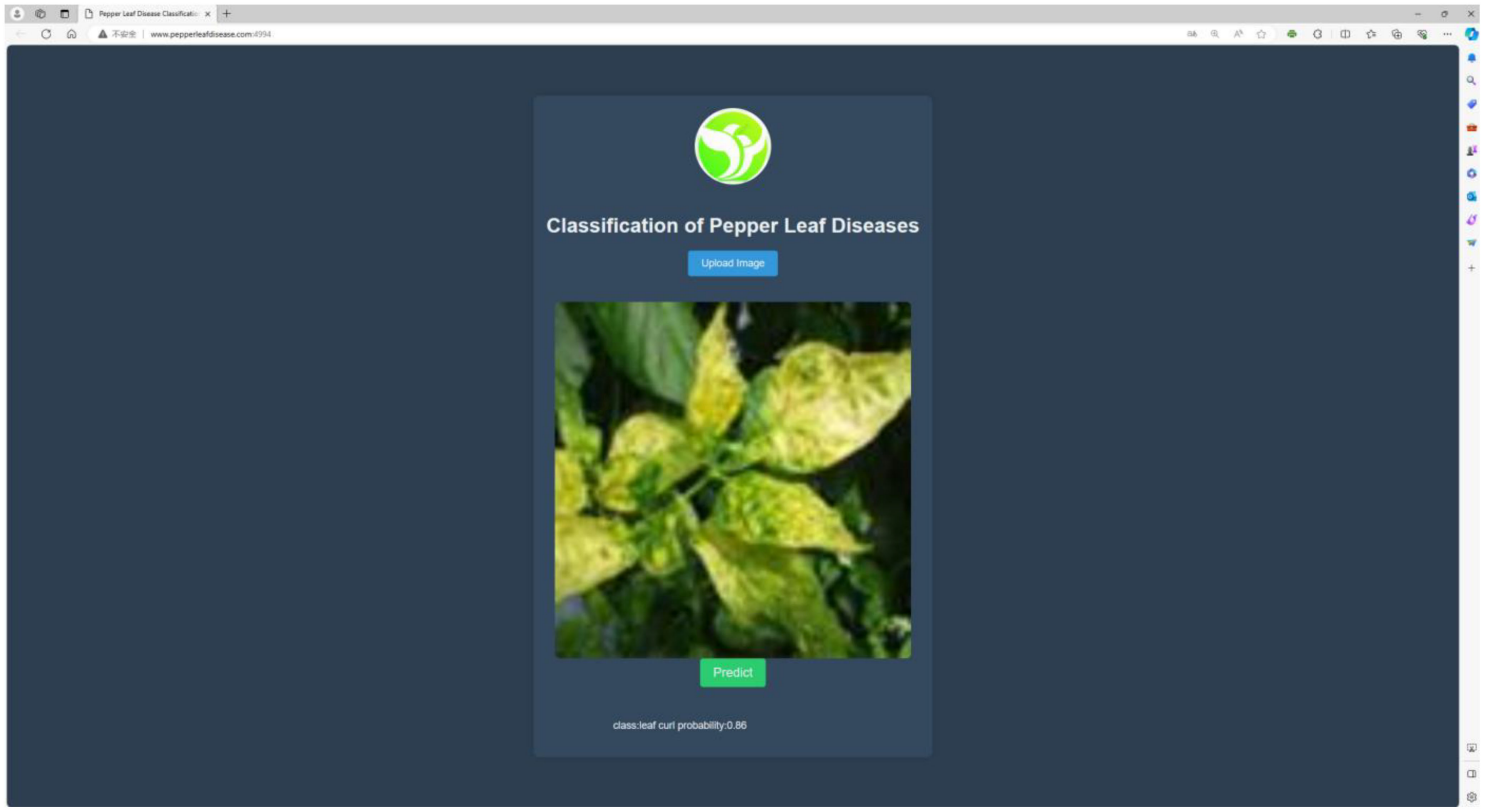
Web page and Test prediction result.

The model is deployed on a web-based platform built using the Flask framework to make it more suitable for practical applications. This system can rapidly and accurately identify four types of diseases in chili plants. The efficient recognition capability assists users in promptly identifying diseases in chili leaves and providing timely solutions, thereby reducing the impact of chili diseases on growth, minimizing losses, and achieving the goals of sustainable agriculture. The website has been successfully deployed on a server, primarily featuring disease image uploading and prediction functionalities for the four types of chili diseases. Users can directly access the website by visiting http://www.pepperleafdisease.com:4994/ (accessed on Dec, 1, 2023). **Figure 14** displays the user interface, and the prediction results of the chili leaf disease system. Users upload images for identification, select the “Predict” option, and receive the recognized chili leaf disease. The classification results are obtained in approximately 260 ms. The design of this chili leaf disease identification system not only addresses the limitations encountered in the practical application of chili disease classification but also alleviates the burden of manual work and associated costs.

## 4 Discussion

In the past, research on plant disease classification primarily relied on machine learning-based methods. However, there were challenges in handling large-scale leaf disease datasets and applications. Therefore, researchers turned to deep learning-based approaches, which integrate features from multiple classical models, significantly improving model performance and better addressing the problem of plant leaf disease classification.

As shown in **Table 7**, recent research has demonstrated significant performance of deep learning in addressing plant leaf disease classification. However, most of these studies focused on specific diseases, such as bacterial leaf spot disease in chili, while overlooking other potential leaf diseases. In contrast, this study comprehensively explored four common leaf diseases in chili and designed effective classification and identification methods. By introducing data augmentation techniques such as random Gaussian noise, random rotation, random brightness, and random occlusion, the performance of the model in chili leaf disease classification was successfully improved. Experimental results indicate that the MCCM deep learning model proposed in this study performed remarkably well in classifying images of healthy chili leaves and the four different diseases, achieving an accuracy of 93.5%. Additionally, we developed a website for chili leaf disease classification, providing an innovative solution for practical applications. Although previous research has made certain progress in methods and results, exploration in practical applications has been relatively limited. Therefore, the contribution of this study lies in proposing a comprehensive classification solution for various chili leaf diseases and developing corresponding tools at the application level, which provides valuable references for further research and practice in the field of plant disease identification.

**Table 7:** Performance comparison of relevant studies.

Literature	Method	Best accuracy	Study object	Application	Date
Wu et al.	MultiModel-VGR	95.34%	chili leaf disease	×	2020
Mathew et al.	YOLOv5	90%	chili leaf disease	×	2023
Mustafa et al.	CNN	99.99%	chili leaf disease	×	2023
Chaitanya et al.	ResNet + CNN	86.1%	chili leaf disease	×	2023
Chen et al.	HSV + CNN	63.26%	chili leaf disease	×	2023
Dai et al.	GoogLeNet-EL	97.87%	chili leaf disease	×	2023
Ours	MCCM	93.5%	chili leaf disease	√	2023

While the MCCM model has shown promising results in classifying chili leaf diseases, there are still areas for improvement. Firstly, despite augmenting the dataset from 500 to 2500 images through techniques like image preprocessing and data augmentation, there may still be challenges related to overfitting or underfitting during training. Secondly, although the optimized MCCM model exhibits high accuracy in chili leaf disease classification, the addition of various modules has increased the demand for computational resources, leading to longer training times. Furthermore, the model tends to misclassify yellowing disease as leaf curl disease. Research analysis revealed that in some cases, yellowing disease and leaf curl disease may exhibit certain similarities in leaf color and texture, especially during the early stages of the disease or under specific environmental conditions. If the MCCM model fails to accurately capture these subtle differences, it may easily misclassify yellowing disease as leaf curl disease. Furthermore, compared to the more effective Swin-Transformer model, the MCCM model can only capture information through local receptive fields and cannot efficiently obtain global information as the Transformer model does when processing sequence data. Therefore, the effectiveness of the MCCM model in distinguishing between the general symptoms of leaf curl disease and yellowing disease may be slightly inadequate. Finally, the chili leaf disease recognition website built on the Flask framework needs enhancements. Currently, the website only supports the recognition of four disease types (leaf curl disease, leaf spot disease, whitefly, and yellowing disease), and its interactive features are relatively basic.

In light of the aforementioned limitations, our next step involves expanding the chili leaf disease dataset to encompass a greater variety of types and a larger number of images (Saleem et al., 2019). Simultaneously, we plan to streamline the model based on practical requirements, reducing redundant connections and parameters to achieve model lightweighting. To address the issue of the model misclassifying yellowing disease as leaf curl disease, it’s necessary to collect high-quality images of both yellowing disease and leaf curl disease to ensure that the model can better extract the subtle differences between the two. Additionally, considering the advantages of Transformer models, further improvements to the model structure could be made to enhance the model’s ability to distinguish between yellowing disease and leaf curl disease. Subsequently, we aim to broaden the website’s applicability, enabling it to recognize additional disease types and enhancing the user experience and interaction methods for more convenient and efficient diagnostic services.

## 5 Conclusions

This study proposes an improved and optimized MCCM CNN model based on the ConvNeXt network for the classification and recognition of diseases in chili plant leaves. The model introduces the MSFFM to enhance sensitivity to features of different sizes and positions, effectively addressing the issue of extracting features of a single size. The overall model performance is optimized by adjusting the position of the MSFFM module in the block and replacing the original GELU activation function and LN with LRELU activation function and BN. Furthermore, the model incorporates MCSAM to strengthen the model’s connection to non-local channels and spatial features, improving the ability to extract useful features. Transfer learning is employed on the Plant Village dataset to obtain pre-trained weights, accelerating the convergence speed of the model, reducing the risk of overfitting, and minimizing training time. Subsequently, the MCCM model is experimentally compared with several models, including Vgg16, ResNet34, GoogLeNet, MobileNetV2, ShuffleNet, EfficientNetV2, ConvNeXt, and Swin-Transformer, on a preprocessed dataset of chili leaf diseases. The MCCM model achieves accuracy, precision, recall, and F1 scores of 93.5%, 91.84%, 94.56%, and 91.68%, respectively. Compared to the original ConvNeXt model, this represents improvements of 1.2%, 1.5%, 1.5%, and 1.5%. Additionally, the study analyzes the model’s performance in different categories of chili leaf diseases using a confusion matrix, confirming its outstanding classification and recognition capabilities. Moreover, the MCCM model exhibits strong performance in the classification and recognition of diseases in corn and rice plants, demonstrating its excellent generalization capabilities.

Given the limited size of the original dataset, various data augmentation techniques, such as random Gaussian noise and random brightness adjustments, were employed to augment the dataset, resulting in a final set of 2500 images. Finally, a user-friendly website for chili leaf disease recognition was developed using the Flask framework. This not only addresses the challenges of practical applications in the classification of chili diseases but also reduces manual efforts and minimizes losses caused by chili diseases.

## Data availability statement

The original contributions presented in the study are included in the article/**Supplementary Material**, further inquiries can be directed to the corresponding author/s.

## Author contributions

DL: Funding acquisition, Methodology, Supervision, Writing – review & editing. CZ: Data curation, Methodology, Software, Writing – original draft, Writing – review & editing. JL: Investigation, Writing – review & editing. ML: Conceptualization, Writing – review & editing. YT: Resources, Supervision, Validation, Writing – review & editing. MH: Supervision, Writing – review & editing.
