# Supplementary material for: MCCM: multi-scale feature extraction network for disease classification and recognition of chili leaves
Source: Front Plant Sci. 2024 May 28;15:1367738. doi: 10.3389/fpls.2024.1367738 (PMC11165206; doi:10.3389/fpls.2024.1367738)
Supplement: Supplementary file 1 [file DataSheet_1.docx]

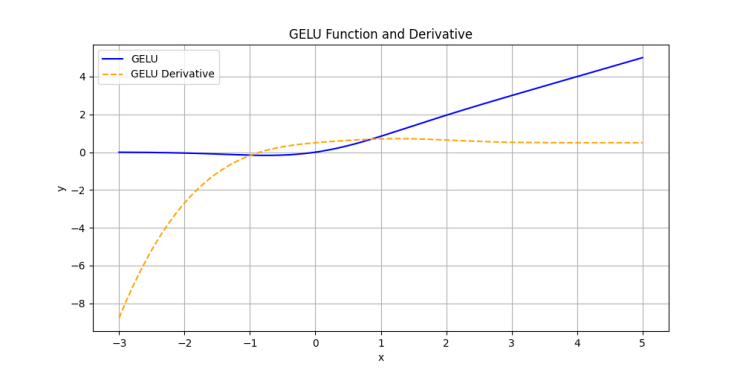
**
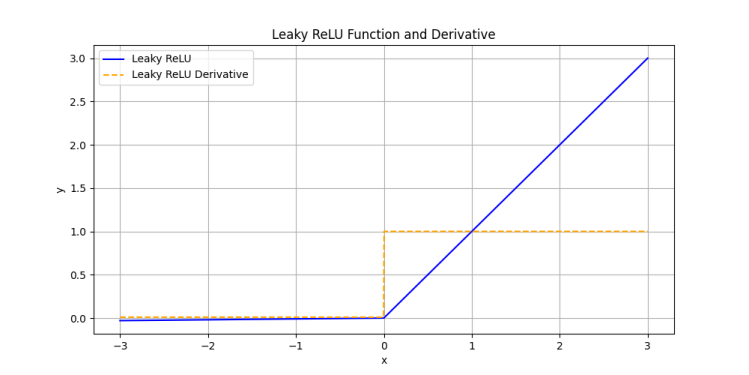
**

(A) (B)

**Figure S1 Activation function image and its derivative image. (A) GELU. (B) LRELU. The properties of GELU and LRELU primitive functions and their corresponding derivatives are compared. The solid blue lines represent the original functions, while the dotted yellow lines represent the corresponding derivatives. The x-coordinate represents the independent variables, and the y-coordinate represents the actual values of the functions and derivatives.**

| **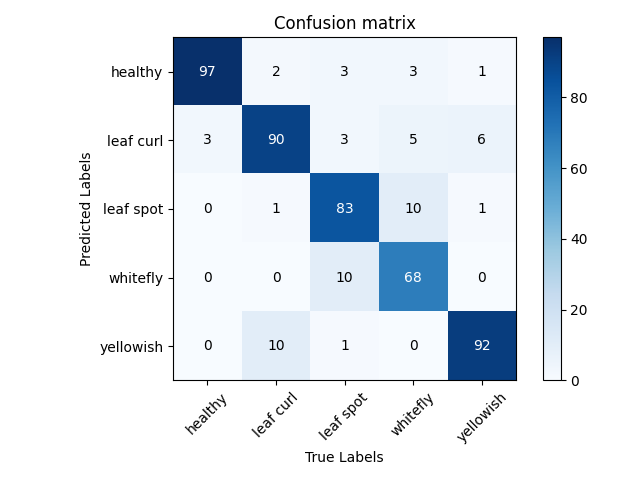** | **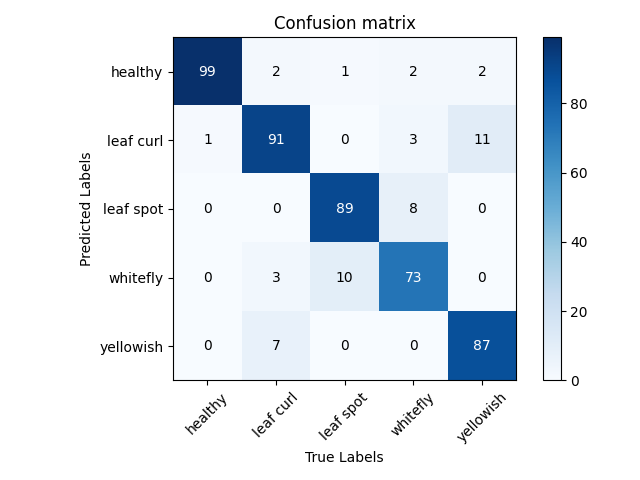** |
| --- | --- |
| **(A)** | **(B)** |
| **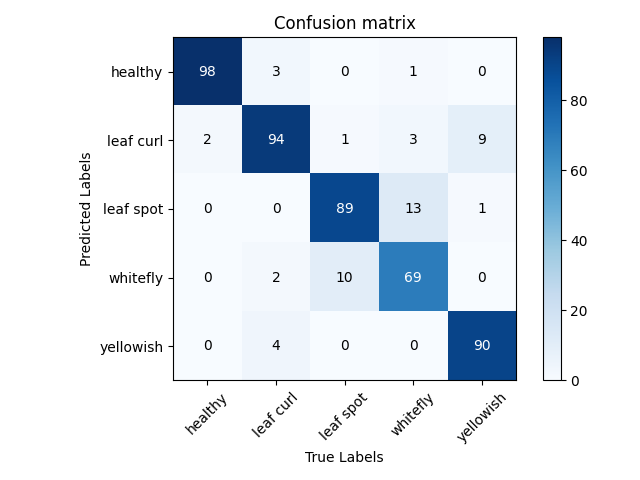** | **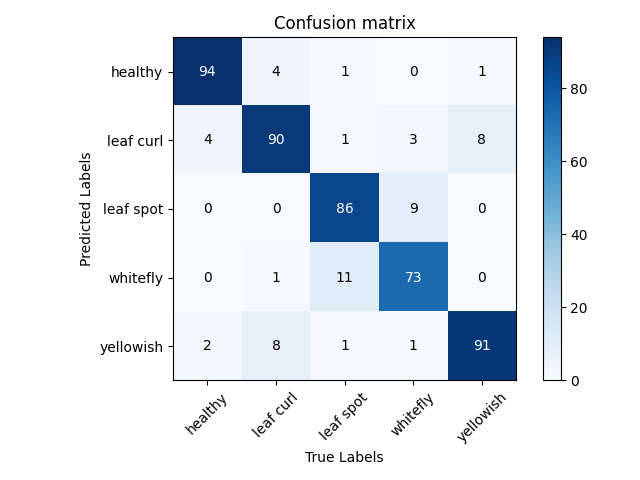** |
| **(C)** | **(D)** |
| **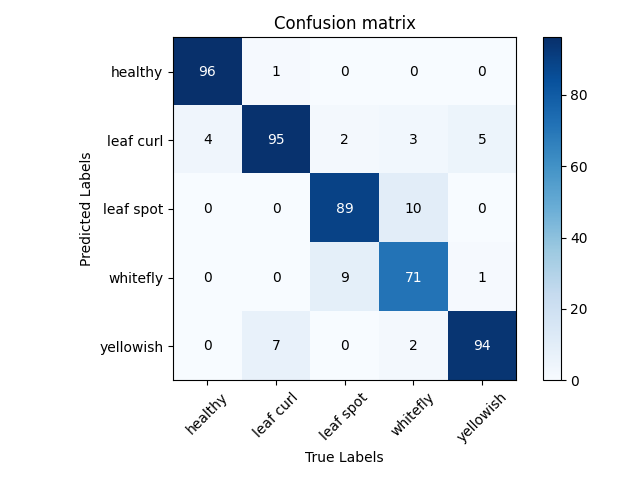** | **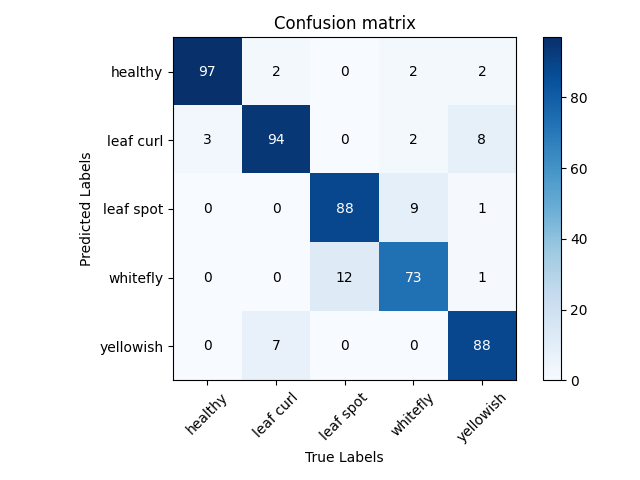** |
| **(E)** | **(F)** |
| **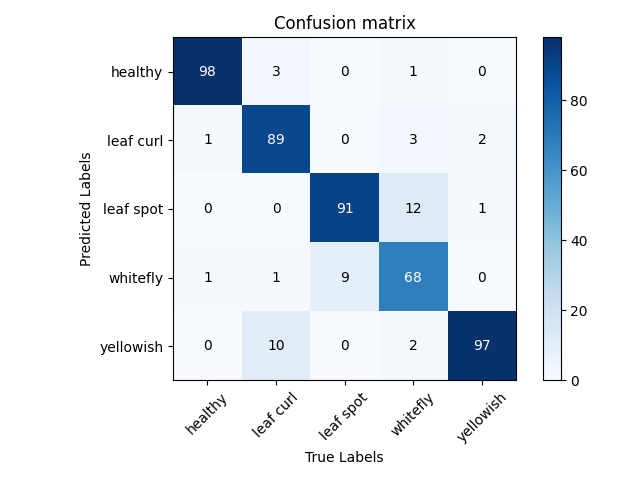** | **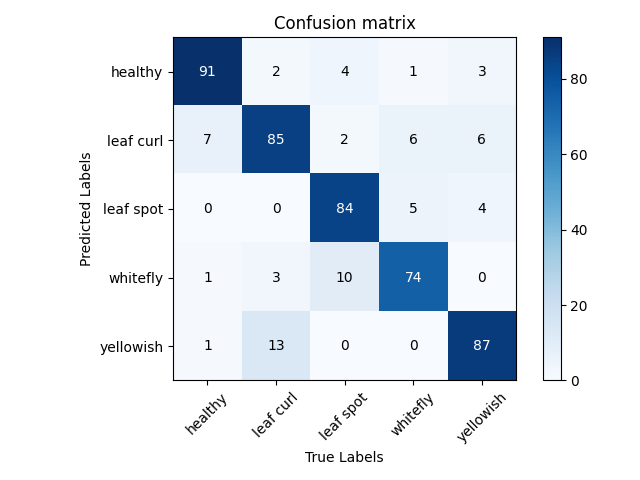** |
| **(G)** | **(H)** |
| **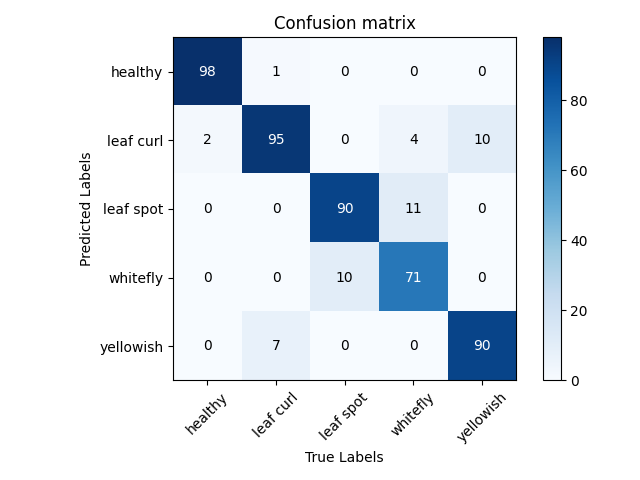** | |
| **(I)** | |

Figure S2 Confusion matrix of classification results of various models on chili leaf disease test set. (A) Vgg16. (B) ResNet34. (C) GoogLeNet. (D) MobileNetV2. (E) EfficientNetV2. (F) ShuffleNet. (G) Swin-Transformer. (H) ConvNeXt-T. (I) MCCM.

**Table S1. Performance comparison of relevant studies.**

| **Literature** | **Method** | **Best accuracy** | **Study object** | **Application** | **Date** |
| --- | --- | --- | --- | --- | --- |
| Wu et al. | MultiModel-VGR | 95.34% | chili leaf disease | × | 2020 |
| Mathew et al. | YOLOv5 | 90% | chili leaf disease | × | 2023 |
| Mustafa et al. | CNN | 99.99% | chili leaf disease | × | 2023 |
| Chaitanya et al. | ResNet + CNN | 86.1% | chili leaf disease | × | 2023 |
| Chen et al. | HSV + CNN | 63.26% | chili leaf disease | × | 2023 |
| Dai et al. | GoogLeNet-EL | 97.87% | chili leaf disease | × | 2023 |
| **Ours** | **MCCM** | **93.5%** | **chili leaf disease** | **√** | **2023** |
